# Supplementary material for: Inter-rating reliability of the Swiss easy-read integrated palliative care outcome scale for people with dementia
Source: PLoS One. 2023 Aug 2;18(8):e0286557. doi: 10.1371/journal.pone.0286557 (PMC10395940; doi:10.1371/journal.pone.0286557)
Supplement: S1 Table — This file contains tabular data for each cluster in a long format. (HTML) [file pone.0286557.s001.html]

Cluster-wise Sociodemographic Statistics.


# Cluster-wise Sociodemographic Statistics.

#### 2023-03-28

Click on a column header to sort the table by that column. Use the
search box on the upper right or filters in the table by specific
values.
