# Supplementary material for: Inter-rating reliability of the Swiss easy-read integrated palliative care outcome scale for people with dementia
Source: PLoS One. 2023 Aug 2;18(8):e0286557. doi: 10.1371/journal.pone.0286557 (PMC10395940; doi:10.1371/journal.pone.0286557)
Supplement: S2 Table — This file shows additional item characteristics for the easy-read IPOS-Dem and complements Table 3. (HTML) [file pone.0286557.s002.html]

Item Characteristics


# Item Characteristics

#### 2023-03-29

The items below are listed in the order they are printed on the
easy-read IPOS-Dem. Click on a column header to sort the table by that
column. Use the search box on the upper right or filters in the table by
specific values. Scroll to the right to see additional descriptive
statistics.

a Removed from analysis.

b Trim = .1

c from the median.

d: Based on a experimental IPOS-Dem Sum Score (NA’s / Do
not know’s were median imputed)
