# Supplementary material for: Inter-rating reliability of the Swiss easy-read integrated palliative care outcome scale for people with dementia
Source: PLoS One. 2023 Aug 2;18(8):e0286557. doi: 10.1371/journal.pone.0286557 (PMC10395940; doi:10.1371/journal.pone.0286557)
Supplement: Attachment — Submitted filename: PeerReview_PONE_D_22_22847.pdf [file pone.0286557.s003.pdf]

Dear authors,

Thank you for submitting your manuscript "Inter-rater and test reliability of the Swiss easy-read Integrated Palliative Care Outcome Scale for People with Dementia" (PONE-D-22-22847) to PloS ONE. This manuscript presents a secondary analysis of a quasi-experimental study in order to delineate different forms of reliability for the IPOS-Dem. Overall, while this manuscript is well prepared I found the overall description of methods to be confusing. Vital information is not presented to understand the measurement schedule and which raters were available when for assessments. For a general readership (like the readership of PloS ONE), there is not enough framing around the issue of measurement in dementia, available measures and assessment in the nursing home setting. The methods are ill-described so that a replication of the study is not possible. I would like to suggest major revisions.

Major issues

Abstract: It would be advisable to term this a secondary analysis of an experimental study. This was not an observational study specifically designed for delineating reliability. Rather, this seems to be an analysis piggy-backing on a larger study. This should be made clear.

I would make it clear in the abstract that this is intended for nursing homes only.

Can you please nuance the results? Or rather - please acknowledge the major problem with the assessment schedule (please see below) in the discussion section. Your conclusions only present one possible explanation for the data. This might be driven to a large part by bias being introduced due to lag in measurement and change in raters for inter-rater reliability assessment. When one does not set up the study in a way that reliability = being free of measurement error can be deduced due to numerous design and rater bias being present in the study, one cannot conclude the unreliability of the measure. Rather, it should be the reduced suitability of the design to determine reliability.

There is a general mismatch of parts in the abstract - the results should not be one sentence when the background section and the objectives take up so much space. The Methods section should also state the sampling mechanism and the full analytical methods.

Background

For a generic journal like PLOS ONE, the background section explains too little about dementia, dementia care (settings, burden of the disease), why assessment is important and which assessment instruments are currently available.

Line 65 f.: This statement is not substantiated or - rather - does not follow. Why should assessments be critical for maintaining quality of life? Surely, the mere assessment of a symptom or problem does not automatically result in this symptom or problem being treated. Joanna Greenhalgh has presented

all the intermediate steps that need to happen for assessment to translate into QOL benefit back in 2000 and 2001. Overall, there are a couple of instances in the background section were these erroneous conclusions are presented without evidence for why this should be the case.

For a background section, I would also like to see a clear description of the setting in which the IPOS-Dem will be used. This study seems to focus exclusively on nursing home. It would be informative for the reader to know about the setting, how care is usually delivered in this setting, how much assessments play a role in this setting and how current assessment methods are challenged. The IPOS-Dem also sits within the realm of patient-centred measures and I was missing an explanation of how the concept of patient-centredness via proxy assessments can figure in the nursing home setting.

What is also missing from the background section is a detailed review of the evidence around assessment/measurement of symptoms and problems in dementia. Clare Ellis-Smith has reviewed the available measures in her systematic review published in PLOS Medicine. It would also pay off to substantiate and prepare the discussion of results of this current study by reviewing reliability studies of other measures (for example, there are reliability studies of the QUALIDEM and other quality of life measures in dementia, also in the nursing home setting (granted - they are rare but they are out there)). It would help greatly to put results into context and one can only do so when knowing the available evidence. Of course, it needs to be pointed out that these other measures have mainly been developed for milder forms of dementia and cognitive impairment. But there are domain-specific (i.e., pain) measures for the nursing home setting. I would give an overview of this wider context in the background section to be able to contextualise and compare/contrast results of this study with the wider literature in the discussion section.

On page 4, you repeatedly describe the IPOS-Dem as a measure to assess the "needs" or "unmet needs" (line 71, line 72) of people with dementia. This is incorrect. According to Bradshaw's definition of types of needs the IPOS-Dem does not measure needs or unmet needs. Rather, I would put the construct more simply as "symptoms and concerns". After all, this is also the official line for the parent measure, the Integrated Palliative Outcome Scale.

Given the generic readership of the journal, it would be good to also contextualise why the IPOS-Dem presents a palliative care approach for dementia. For this, it would be important to refer to the substantial suffering and symptom burden in dementia patients. I'd like to recommend reviewing Elizabeth Sampson's work (for example, doi: 10.1186/s12877-017-0523-3) and Jenny van der Steen's work (nursing home sector in the Netherlands). Again, there haven't been many studies on palliative care/end of life/symptom burden aspects within dementia care but the few out there could be mentioned as evidence in the background section. Currently, both the background and discussion sections are very "light" in terms of literature.

Methods

It is curious to see a direct contradiction in the article on subsequent pages when it comes to the description of the study design. On page 4, it is explained that this study sits within the stepped-wedge RCT (ref 13). Yet, on page 5 it is introduced as an observational study. It also mentions the cluster design normal within the experimental realm of study designs. First, I would term this a secondary analysis of said cluster/stepped-wedge trial. The term observational study should be left for studies truly observational (non-experimental) in nature. For an observational longitudinal study, I would expect this to be a cohort study with an exposed and a non-exposed group. This is not the case. It also helps with the aspect of adequate power (please see below). However, the clustered nature should ideally be accounted for in the analysis unless you can argue consistently why this is not necessary. It could be acknowledged via generalizability theory (more on this later).

In this regard, I would advise using the COSMIN checklist instead of a reporting guideline geared towards measurement properties.

Data collection: Please clearly describe whether data was captured on electronically or on paper. You repeatedly talk about REDCap, a "survey" being created (line 120), assessments being made but these descriptions are not clear in the sense that they could be replicated. The data collection section should unambiguously describe the methods and processes used for collecting data.

Table 1 - could you please clearly describe what these measurement time points T1, T2, and T3 are? When were these assessed? Please give exact timings.

Line 138: These are not the correct descriptors for the answer scale's endpoints on the IPOS-Dem. It should also be made clear that each response option on the answer scale has a descriptor, not only the endpoints of the answer scale.

General design issue:

Now, for assessing inter-rater reliability, ideally the same pair of raters should independently rate the same patient at a time point. From the description given in the methods section, it seems that this has not always been the case. Am I correct in assuming that not the same raters were used for each patient for inter-rater reliability assessment?

The same question can be asked of the description of the test-retest reliability design. For true test-retest reliability, the same person should rate the same patient over time. It is unclear from the description whether this was indeed the case. This is the more vital given that an "external" change criterion in form of the global rating of change question was used to determine change or stability. If different persons rated the GRC from timepoint to timepoint, this would result in measurement error being introduced stemming from different raters.

Two aspects need further argumentation: (a) Why was the GRC constructed with three answer options? There is quite a lot of methodological literature around the construction of a global rating of change and the number of answer options. I can understand the simplistic approach of worsened - the same - better. I just think it needs careful argumentation why this is appropriate. Now, for a GRC to work properly within a stability/change construct validity or reliability design, it needs to be argued that the criterion itself is free of measurement error (GRCs as external criteria essentially

constitute a version of criterion construct validity (see Henrica de Vet and coauthors in Measurement in Medicin, Cambridge Uni Press 2011)). They cannot act properly as external criteria once they are measurement with measurement error. In this case, the determination of test-retest reliability is tipping one measurement-prone measure against another measurement-prone measure. This will result in potentiation of measurement error and in the attenuation of correlation/agreement measures like the ICC.

(b) Why was a period of one month between assessment deemed appropriate for stability being measured? There are no pre-fabricated answers to this one (and barely any literature). However, I would think that people with dementia in nursing homes change more quickly. Why was a more frequent measurement schedule disregarded? I'd like to see some reasoning on this.

Line 152: population

This is a minor issue but this section should sit with the sample section. The normal structure of a methods section is study design (plus ethics) - population/setting & sampling mechanism & sample (eligibility criteria, sampling methods) - data collection (instruments) - data analysis.

Line 149 f: Please describe clearly which sociodemographic or clinical information was taken from the RAI.

Analysis

Reliability assessment really only makes sense once the content validity and construct validity has been determined. You can reliably measure the wrong thing. I'd like to see a careful argumentation why you deem content and construct validity of the measure to be given to a degree that reliability assessment makes sense. Reliability means being free of measurement error. This measurement error can stem from random error, but it can also be introduced due to systematic error (bias). Systematic error itself can be introduced through invalidity.

It would also need reasoning around why the assessment of reliability is important for the measure.

Line 179: Please revise the sentence around the scores being treated as continuous. First, this guidance is for the parent measure, not the IPOS-Dem. In contrast to the IPOS-Dem, factor analytical and Rasch measurement work has been done for the IPOS to deem whether a sum score and subscale scores are appropriate. The answer scale itself remains ordinal in nature. It is inappropriate and wrong to state that the answer scale constitutes continuous data. It also gives the wrong impression that this is coming from the developers.

Treating the raters as random is a further pointer towards different raters being used for the single assessments. While this is alright as such, it will result in lower ICC estimates. I would recommend a clear discussion on this issue in the discussion section. Since you are going for variance composition anyway, you could actually clearly determine the standard error of measurement and further explore how large each source of variance (error) is. I was missing this more nuanced presentation from the

results and discussion sections. This would also answer the question how much variance can be attributed to the change in raters and how much this source of variation has attenuated the ICCs.

Likewise, I would like to see an assessment of how well the GRC functioned to determine change or stability in the people with dementia being rated. My suspicion is that low test-retest reliability estimates may be due in parts to the invalidity of the GRC to determine stable people with dementia being rated. I would recommend exploring this aspect in the results. Henrica de Vet describes some methods for determining the GRC's validity in their chapter on responsiveness/longitudinal construct validity.

Reliability estimates are affected by floor (and ceiling) effects of measures. From Table 4, it later becomes apparent that many items suffer from large proportions in the lowest or the two lowest answer categories (answer option 0 and 1). Yet, no analytical measures are being taken to determine its effect on the reliability estimates.

Sources of unreliability could be explored within the generalizability framework. I'd leave it to the authors whether they want to run this re-analysis of the data. If they decide to not run this, I advise carefully arguing why this angle is not pursued and also carefully arguing this limitation in the discussion section, drawing on methodological literature to do so. A couple of reliability studies of dementia measures have also struggled with this aspect. Here, doing a wider literature search around reliability studies in dementia will help locate appropriate cases of studies for comparison.

Sample size calculation

It has not been made clear that this constitutes a secondary data analysis of a stepped-wedge trial. As such, I understand that it was not possible to directly power this reliability study a priori. However, this does not absolve from thinking carefully through power issues. Please add a full sample size calculation working through a full assessment how the given sample with the given number of raters under a random-effects model for reliability determination and given a desired level of precision in estimates allows you to delineate ICCs and coefficients of a minimum size. Given the amount of measurement error from changing raters across measurement points and the unclear assessment schedule in general, also from having a measurement-prone external change criterion, may severely limit the maximum ICC achievable given the sample size (raters and patients). I would therefore shift the discussion around sample size from an a priori powering to a post-hoc determination of power and how low power may affect the ICC estimates. I was also missing a discussion of this limitation in the discussion section. Please work through these issues in a clear and open way.

Bland-Altman plots: These only work on the total scores of measures. As of today, there has not been a study demonstrating the factor validity of the measurement model of the IPOS-Dem. Summing all scores to form a total score is therefore not supported by the available psychometric evidence. To do so in this analysis constitutes a preliminary and exploratory practice and should be termed as such. The BA plot also demonstrates the integer nature of the sum score. There are methods to correct for this but you do not need to go there. Terming this analysis as exploratory in nature and clearly

describing this limitation (of not currently being safe to sum all items) in the discussion section would be enough in my view.

Results

As much as a find Sankey diagrams cool (and I am very fond of playing with them in my data visualisations), I do not think that Figure 1 transports information in an unambiguous way. Without diving into this, it is very hard to understand (a) what the "n" means - are these people with dementia or raters?, (b) the reasons for why so many are dropping out, (c) whether the GRC = change = n = 91 were people that reported a change somewhere or for whom a change was reported at both T1 and T2 and T3, (d) whether further missing data occurred beyond T1. I think this may work better in a proper flowchart. In a flowchart, please also add (a) when T1, T2 and T3 were taken and ideally give median number of days between time points to show the assessment schedule in full, (b) also note the number of raters per time point and note how many people had different raters from time point to time point.

Line 202: I think you mean that this nursing home decided to withdraw from the study, not "drop out".

Line 203: Do you mean you oversampled? "A surplus in sample size" at T0 is hard to understand. Please state clearly: (a) how many nursing homes took part, (b) how many people with dementia per nursing home and how many raters per nursing home, (c) how many raters per time point per nursing home rated the same persons with dementia.

Line 210: The heterogeneity in assessment times is such an important limitation for the findings that it should be presented clearly in the main part of the paper, not the appendix. Please be clear about the measurement schedule, the number of persons with dementia, the number of raters and change of raters. This point refers to the whole manuscript.

Line 222: This does not present all item characteristics which would normally be reported in item analysis (item difficulty, item variance / homogeneity, item-total correlation etc. --> please see "scale analysis", for example Moosbrugger & Kelava. Testtheorie und Fragebogenkonstruktion. Springer). I would describe and add these methods to the analysis section. I would also clarify which item characteristics have been determined. Please also clearly define floor and ceiling effects and give the appropriate reference for a cutoff in the methods section.

Table 4: The items are not listed in the order in which they occur in the IPOS-Dem. Please clarify or reorder them. Please also give more results by clearly naming those items that exhibit floor/ceiling effects or show problematic means.

You may want to add variance decomposition to understand the sources of variation and error that are present in the results. It would be worthwhile working through a determination of the standard error of measurement and also understanding the validity of the GRC as a prerequisite for determining test-retest reliability.

Discussion

I have given numerous pointers for a restructuring of the discussion section. The current discussion section, although addressing some limitations of the study, does not address some of the more important limitations upfront. I was also missing a contextualising of results via comparisons to similar reliability studies of different measures in dementia care. There should also be a wider discussion on the issue of construct validity of the IPOS-Dem for the nursing home population. There is also a discrepancy between the statement that the IPOS-Dem "can be used by frontline staff" to assess the concerns and then stating that they will do so unreliably. The first statement is not supported by the findings of the study, mainly because validity of the IPOS-Dem has not been assessed in the current article. This is confusing different measurement properties of the instrument.

Page 19: While the IPOS-Dem might be easy to understand, the unreliability of the measure points towards not enough context being given for a rater to make the assessment. This could be addressed by incorporating descriptors around items. Without having the IPOS-Dem in Swiss German presented to the reader in the appendix, the reader is asked to take the assertion on line 275 ff at face value. I would (a) substantiate this claim and (b) clearly describe the changes in the measure itself that are necessary to address the problems with content and construct validity.

Page 20, line 297: Please delete the first sentence in this section starting on line 297. This is an incorrect statement (and a very dangerous one to make).

The discussion from page 20 onwards seemingly veers into a discussion for a different type of study. A lot of musings on the implementation of the measure in nursing homes seem to be offered. I was asking whether this bit was taken from the larger stepped-wedge RCT? It seems to be poorly linked to the reliability work and findings presented in the current manuscript. This was also apparent in the conclusion section in which new comparison evidence was cited and introduced, again pertaining to the implementation and not the reliability of assessments in dementia care.

I would like to advise rewriting the discussion section to incorporate the recommendations given above. It may help to consult the article by Docherty & Smith (DOI: 10.1136/bmj.318.7193.1224) on how to structure a discussion section.

Minor issues

There are a couple of minor mistakes (i.e., line 96 within a stepped-wedge and without the comma before "with"). There are also instances of odd word choices which could be checked.

You may consider using jittering in the Bland-Altman plots (there are pro and con arguments for this, maybe present both?).
