# Supplementary material for: Inter-rating reliability of the Swiss easy-read integrated palliative care outcome scale for people with dementia
Source: PLoS One. 2023 Aug 2;18(8):e0286557. doi: 10.1371/journal.pone.0286557 (PMC10395940; doi:10.1371/journal.pone.0286557)
Supplement: Attachment — Submitted filename: Response_to_reviewers.html [file pone.0286557.s004.html]

Response to Reviewers


# Response to Reviewers

#### 2023-04-14

Thank you for all your helpful feedback. Due to the amount of
commentary we decided to display our response in the Table below. The
reviewer comments are grouped by default. This is an interactive table,
please also use the search field or column headers to find your comment
and our responses/changes.
